# Outcomes of Intracranial Haemorrhage in Patients Taking Direct Oral Anticoagulants or Vitamin K Antagonists: A Seven-Year Single-Centre Retrospective Analysis

**DOI:** 10.3390/clinpract16040079

**Published:** 2026-04-18

**Authors:** Mallika Sathitwat, Surat Tanprawate, Atiwat Soontornpun, Chayasak Wantaneeyawong, Chutithep Teekaput, Nopdanai Sirimaharaj, Angkana Nudsasarn, Chatree Chai-Adisaksopha, Kitti Thiankhaw

**Affiliations:** 1 Division of Neurology, Department of Internal Medicine, Faculty of Medicine, Chiang Mai University, Chiang Mai 50200, Thailand; 2 The Northern Neuroscience Center, Faculty of Medicine, Chiang Mai University, Chiang Mai 50200, Thailand; 3 Division of Haematology, Department of Internal Medicine, Faculty of Medicine, Chiang Mai University, Chiang Mai 50200, Thailand

**Keywords:** intracranial haemorrhage, direct oral anticoagulant, vitamin K antagonist, outcomes

## Abstract

**Background:** The clinical outcomes of patients with intracranial haemorrhage (ICH) whilst using direct oral anticoagulants (DOACs) and vitamin K antagonists (VKAs) are uncertain. This study aimed to assess outcomes and management in patients receiving DOACs compared with those receiving VKAs. **Methods:** In this retrospective study, patients hospitalised during the period from 1 January 2017 to 31 December 2023 for traumatic and non-traumatic ICH and using oral anticoagulants (OACs) were included. The primary outcomes were mortality and functional outcomes, as measured by the modified Rankin Scale (mRS) during admission and 90-day follow-up. ICH management and complications were studied and compared between the two OAC groups. **Results:** A total of 171 eligible patients were included, comprising 24 patients on DOACs and 147 patients on VKAs. Patients receiving DOACs were older (79.1 vs. 66.8, *p* < 0.001) and had a higher proportion of traumatic ICH (75.0% vs. 46.3%, *p* = 0.009) than those receiving VKAs. In-hospital and 90-day outcomes were not statistically different between the two groups, with an adjusted odds ratio (aOR) of 1.30 (0.39–4.36) for in-hospital mortality, *p* = 0.67, and an aOR of 0.89 (0.33–2.41) for mRS 0–2 at 90 days, *p* = 0.83. In total, 81.3% of patients received at least one reversal agent; fresh frozen plasma was commonly used in the VKA group (78.9% vs. 33.3%, *p* < 0.001), whereas prothrombin complex concentrate was significantly prescribed in patients with DOAC-associated ICH (29.2% vs. 3.4%, *p* < 0.001). **Conclusions:** Patients with DOAC-associated ICH had comparable in-hospital and long-term clinical outcomes to those with VKA use.

## 1. Introduction

Intracranial haemorrhage (ICH) is a life-threatening complication of oral anticoagulation, associated with high mortality and disability [1]. Vitamin K antagonists (VKAs) substantially increase both the risk and severity of ICH [2]. Direct oral anticoagulants (DOACs) were introduced as safer alternatives, demonstrating lower rates of spontaneous ICH in randomised trials and real-world studies [3].

Despite these preventive advantages, registry data suggested that significantly smaller haematoma volume, haematoma expansion, and a lower mortality rate were observed under DOACs compared to VKAs [4,5,6,7]. Another study highlighted that whilst clinical and experimental studies suggest a less devastating profile under DOACs [8], DOAC users are still at risk of developing ICH, and studies on the outcomes of DOAC-associated ICH have had inconsistent results; for example, some studies report smaller initial haematoma volume, less hospital mortality, and better discharged functional outcome, whilst others show similar outcomes between groups [9,10,11,12]. These findings remain heterogeneous due to methodological limitations, impractically small sample sizes, or critical imbalances in baseline parameters and selection biases [13].

The data on functional outcomes are typically limited and inconsistent [12]. Evidence on reversal strategies and surgical outcomes remains limited, as most randomised surgical trials excluded patients who were anticoagulated [14,15]. Asian data, in particular, are scarce, despite potentially different demographic and management profiles. This study, therefore, aimed to compare outcomes and management strategies for DOAC- versus VKA-associated ICH in a real-world Asian tertiary-care setting.

## 2. Methods

### 2.1. Study Design and Patient Selection

**Figure 1:**
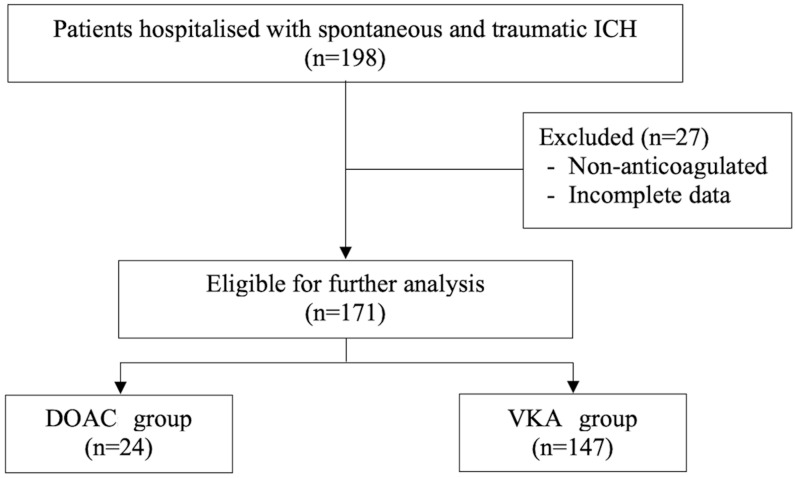
Flowchart of patient selection. DOACs, direct oral anticoagulants; ICH, intracranial haemorrhage; VKAs, vitamin K antagonists.

This single-centre retrospective cohort study included patients hospitalised with spontaneous and traumatic intracranial haemorrhage whilst receiving oral anticoagulation at Maharaj Nakorn Chiang Mai Hospital, a tertiary centre and university-affiliated hospital of Chiang Mai University, between 1 January 2017 and 31 December 2023. Data were assessed for research purposes from 20 March 2024 to 19 March 2025. Eligible patients were aged ≥ 18 years with radiologically confirmed ICH. Patients with known underlying structural or macrovascular lesions (e.g., arteriovenous malformation, dural arteriovenous fistula, cavernomas, or cerebral aneurysms) or cerebral ischaemia with haemorrhagic transformation were excluded. The flowchart of the included participants is illustrated in Figure 1.

### 2.2. Data Collection

Demographic and clinical characteristics were reviewed and extracted from the electronic medical record (EMR). Baseline variables included age, sex, height, weight, comorbidities (hypertension, dyslipidaemia, diabetes, previous cerebrovascular diseases, coronary heart disease, congestive heart failure and chronic kidney disease), admission blood pressure, indication for anticoagulation (non-valvular atrial fibrillation (AF), venous thromboembolism, valvular heart disease (VHD), or other/unknown), and concurrent use of antiplatelet therapy (aspirin, clopidogrel, cilostazol, or ticagrelor). Neurological status was assessed using the Glasgow Coma Scale (GCS) and modified Rankin Scale (mRS) on admission.

ICH characteristics and haematoma location were collected, and the Cerebral Haemorrhage Anatomical RaTing inStrument (CHARTS) was utilised for spontaneous intracerebral haemorrhage to map the anatomical location into lobar, deep and infratentorial, or uncertain [16]; haematoma volume was calculated using the ABC/2 method [17]. Laboratory data included routine complete blood count, blood urea nitrogen, creatinine, liver function tests, and coagulation profile. Management strategies were recorded, consisting of neurosurgical interventions and the use of reversal agents, including vitamin K, tranexamic acid, fresh frozen plasma (FFP), prothrombin complex concentrate (PCC), idarucizumab, and recombinant factor VIIa (rFVIIa).

### 2.3. Outcomes

The primary outcomes were all-cause mortality and functional status, measured by the mRS during hospitalisation and at 90 days, which were further classified into excellent functional outcome (mRS 0–1), good functional outcome (mRS 0–2), and independent ambulation (mRS 0–3). Secondary outcomes included management strategies (use of reversal agents and neurosurgical interventions), in-hospital thrombotic complications, and length of stay (LOS), which were compared between patients receiving DOACs and those receiving VKAs.

### 2.4. Statistical Analysis

Categorical variables were reported as frequencies and proportions and compared using the Pearson χ^2^ or Fisher’s exact test, as appropriate. Continuous variables were expressed as mean with standard deviation (SD) or median with interquartile range (IQR) and compared using Student’s *t*–test or the Mann–Whitney U test, depending on data distribution. The association between study outcomes and use of oral anticoagulant (OAC) were investigated using univariable and multivariable logistic regression analyses after adjustment for confounders, including elderly age (greater than 60 years), hypertension, aetiology of ICH, volume of intracerebral haemorrhage, treatment, and indication of oral anticoagulant, and demonstrated as crude or adjusted odds ratio (OR) with their 95% confidence interval (CI). Two-sided *p* values < 0.05 were considered statistically significant. All statistical analyses were performed using licenced Stata statistical software version 16.1 (Stata Statistical Software: Release 16.1, Stata Corporation, College Station, TX, USA, 2019).

## 3. Results

A total of 171 patients with oral anticoagulant-associated intracranial haemorrhage were included and analysed, comprising 24 patients (14.0%) on DOACs and 147 patients (86.0%) on VKAs (Figure 1). The mean age of the included study population was 68.5 ± 12.7 years, and 48.5% were male. Patients receiving DOACs were significantly older (79.1 ± 9.6 vs. 66.8 ± 12.4 years, *p* < 0.001), had more hypertension (79.2% vs. 48.3%, *p* = 0.007) and dyslipidaemia (54.2% vs. 32.0%, *p* = 0.04) when compared with VKA patients, whilst the prevalence of other comorbidities including diabetes, coronary heart disease, congestive heart failure, previous stroke and chronic kidney disease did not differ significantly between the two groups. Admission mRS, GCS, and concomitant use of antiplatelet therapy did not differ statistically between the two OAC groups. The ATRIA (Anticoagulation and Risk Factors in Atrial Fibrillation) Bleeding Risk Score was significantly higher in patients prescribed DOACs (5 (3, 6) vs. 3 (3, 5), *p* = 0.04). AF was a primary indication for the use of DOACs, whilst VKAs were significantly prescribed as OAC for patients with VHD (Table 1).

**Figure 2:**
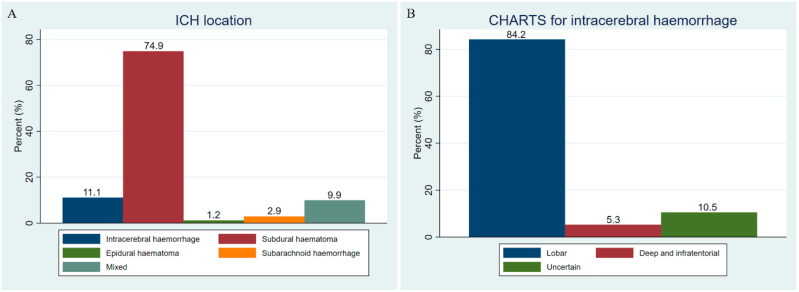
ICH information. (**A**) ICH location. (**B**) CHARTS for intracerebral haemorrhage. CHARTS, Cerebral Haemorrhage Anatomical RaTing inStrument; ICH, intracranial haemorrhage.

On ICH characteristics and management, approximately half of all ICH cases were traumatic ICH, with a higher proportion in the DOAC group (75.0% vs. 46.3%, *p* = 0.009). Subdural haematoma (SDH) was the most frequent type of ICH (74.9%), followed by intracerebral haemorrhage (11.1%), mixed location (9.9%), subarachnoid haemorrhage (SAH) (2.9%), and epidural haematoma (EDH) (1.2%). Mixed-location haemorrhage was more frequent in DOAC patients (20.8% vs. 8.1%). Among intracerebral haemorrhage, lobar distribution predominated (84.2%), and the mean intracerebral haematoma volume tended to be larger in the DOAC group, compared with VKA patients (13.9 ± 47.3 vs. 4.0 ± 17.0 mL, *p* = 0.06, and 58.3 (37.5–82.6) vs. 27.9 (15.9–49.2), *p* < 0.001, in geometric means using log transformation) (Table 2 and Figure 2).

Neurosurgical interventions were performed in nearly half of the study’s population (51.9%), with statistical differences between the two groups (*p* = 0.03). In DOAC patients, external ventricular drainage (29.2%) and burr hole drainage (20.8%) were common procedures, whereas intracerebral haemorrhage removal was more frequent in VKA patients (25.9% vs. 8.3%) (Table 2). Reversal strategies also differed substantially. Vitamin K (41.5% vs. 4.2%, *p* < 0.001) and FFP (78.9% vs. 33.3%, *p* < 0.001) were predominantly administered to VKA-associated ICH patients. Conversely, DOAC patients were more likely to receive PCC (29.2% vs. 3.4%, *p* < 0.001) or idarucizumab (20.8% vs. 0%, *p* < 0.001). Tranexamic acid and rVIIa were infrequently used without significant differences (Table 2). The median hospital length of stay was 11 days (IQR, 6–18), with no statistical difference between groups (10 days (5, 14) vs. 11 days (6, 18), *p* = 0.30). Thrombotic complications were observed only among VKA patients (4.8%), including myocardial infarction (0.7%), stroke (2.0%), and other events (2.0%). No thrombotic events were reported in the DOAC group (Table 2 and Supplementary Table S1).

**Figure 3:**
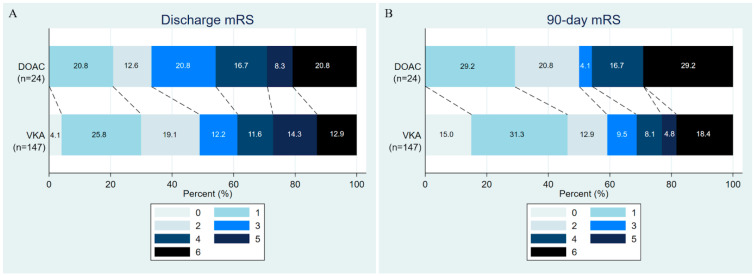
Functional outcomes of the study population. (**A**) Discharge mRS and (**B**) 90-day mRS. mRS, modified Rankin Scale. DOAC, direct oral anticoagulant; mRS, modified Rankin Scale; VKA, vitamin K antagonist.

Regarding clinical outcomes, in-hospital mortality was 20.8% among patients treated with DOACs, compared with 14.3% among those treated with VKAs. After adjusting for potential confounders, including age, hypertension, treatment strategies, and indication of oral anticoagulant, the difference was not significant (adjusted OR, 1.30; 95% CI, 0.39–4.36; *p* = 0.67). Independent ambulation at discharge was achieved in 33.3% vs. 49.0% (adjusted OR 0.88; 95% CI 0.32–2.40; *p* = 0.80). At 90 days, mortality was 29.2% in the DOAC group versus 19.1% in the VKA group (adjusted OR 1.20; 95% CI 0.41–3.53; *p* = 0.74). Functional outcomes were also comparable: mRS 0–1 was achieved in 29.2% vs. 46.3% (adjusted OR 0.83; 95% CI 0.29–2.34; *p* = 0.72), and mRS 0–2 in 50.0% vs. 59.2% (adjusted OR 0.89; 95% CI 0.33–2.41; *p* = 0.83) (Table 3 and Figure 3).

To further contextualise our findings, a post hoc power analysis was performed based on the observed sample sizes and event rates (Supplementary Table S2). The estimated statistical power varied across outcomes, with higher power observed for functional endpoints, including 90-day mRS 0–1 (0.63) and independent ambulation at discharge (0.56).

To further explore the potential impact of ICH aetiology, stratified analyses were performed according to traumatic and non-traumatic ICH (Supplementary Table S3). Across both subgroups, there were no statistically significant differences in clinical outcomes between DOAC and VKA users. In the non-traumatic ICH subgroup, the direction of effect estimates generally favoured DOACs for mortality outcomes, including in-hospital mortality (OR 0.65; 95% CI 0.03–12.5; *p* = 0.77) and 90-day mortality (OR 0.46; 95% CI 0.02–8.70; *p* = 0.60). Functional outcomes were also comparable, with no statistically significant differences observed for independent ambulation at discharge or 90-day mRS categories. Similarly, in the traumatic ICH subgroup, outcomes remained consistent between groups, with no significant differences in in-hospital mortality (OR 1.67; 95% CI 0.53–5.32; *p* = 0.38), 90-day mortality (OR 1.92; 95% CI 0.66–5.58; *p* = 0.23), or functional outcomes.

## 4. Discussion

The main findings of this study revealed no significant differences in short-term and long-term mortality, or functional outcomes, including independent ambulation and favourable functional outcomes, between DOAC- and VKA-associated ICH patients after adjustment for potential confounders. Although DOAC patients were older and more frequently had comorbidities and presented with traumatic ICH, their outcomes were comparable to those of VKA patients.

Our findings are consistent with prior studies, which demonstrated that DOACs are associated with lower early mortality and smaller haematomas compared with VKAs [4,5,6,7]. However, once ICH occurred, both groups experienced poor long-term outcomes [12,18,19]. Additional insights from an Asian registry similarly observed lower early mortality in DOAC patients compared with VKAs, but no difference in 90-day mortality or functional outcomes [20,21].

Focusing on elderly and traumatic ICH populations further expands these observations. A systematic review of 11 studies involving 4991 elderly patients with traumatic brain injury (TBI) revealed comparable outcomes between VKAs and DOACs in terms of intracranial haemorrhage, morbidity, and mortality [22]. Additionally, retrospective studies including 700 elderly patients with TBI found that DOAC use was not associated with increased morbidity or mortality. Moreover, only advancing age and a lower GCS score were associated with higher mortality [23]. In our cohort, DOAC patients were older and more frequently presented with traumatic ICH, yet still experienced outcomes comparable to the VKA group. This may reflect the relatively safer bleeding profile of DOACs, but also illustrates that once ICH develops, outcomes converge across drug classes.

Functional outcomes in our study did not differ between ICH associated with DOACs and that associated with VKAs. These findings are consistent with contemporary registry data and pooled analyses demonstrating that long-term disability after anticoagulant-associated intracerebral haemorrhage is primarily determined by baseline neurological severity rather than anticoagulant class [24]. Similarly, a retrospective cohort study of patients with ICH associated with oral anticoagulation in three tertiary-care hospitals showed no significant differences in ICH outcomes between those receiving DOACs and those receiving VKAs [25]. In addition, a previous international collaborative multicentre pooled analysis included 500 patients with intracerebral haemorrhage, demonstrating that baseline haematoma volume, 90-day mortality, and functional outcome were comparable between DOAC and VKA groups [12]. Collectively, these data reinforce that the anticoagulant class is not an independent determinant of recovery once ICH has occurred.

Importantly, whilst the number of patients in the DOAC group was smaller than in the VKA group, this distribution reflects real-world prescribing patterns in our setting and provides clinically relevant insight into contemporary practice. The post hoc power analysis demonstrated moderate statistical power for functional outcomes, particularly independent ambulation at discharge and 90-day mRS 0–1, whilst power for mortality outcomes was lower due to relatively small event numbers. Notably, the consistency in direction and magnitude of effect estimates across all outcomes, including adjusted analyses, supports the overall robustness and internal validity of our findings. These observations suggest that the comparable outcomes between DOAC- and VKA-associated ICH are unlikely to be solely explained by limited statistical power. Nonetheless, this study may be less sensitive to detecting small effect sizes, and larger, adequately powered studies are warranted to further validate these findings.

Further stratified analyses according to ICH aetiology demonstrated consistent results between traumatic and non-traumatic subgroups, with no significant differences in mortality or functional outcomes between DOAC and VKA patients. The direction and magnitude of effect estimates remained broadly similar across strata, supporting the stability of the primary findings. Although traumatic and spontaneous ICH differ in underlying mechanisms and clinical management, these results suggest that anticoagulant class does not substantially modify outcomes once haemorrhage has occurred. This reinforces the applicability of the pooled analysis and aligns with real-world clinical scenarios in which both aetiologies coexist within anticoagulated populations.

Reversal strategies differed between groups in our study, with vitamin K and FFP primarily administered to VKA patients, whilst PCC and idarucizumab were more commonly used among DOAC patients. Consistent with clinical practice, where PCC-based reversal is well established for VKA-associated ICH but remains variable for DOACs [26,27]. Evidence from the RADOA registry (Reversal Agent Use in Patients Treated with Direct Oral Anticoagulants or Vitamin K Antagonists) supports this treatment pattern: PCC was administered in approximately 70% to 90% of VKA-associated ICH, whereas its use in DOAC-associated ICH was less consistent [4]. The infrequent use of PCC observed in our study may partly reflect contextual factors such as limited access and financial considerations rather than clinical necessity for reversal practice. However, DOAC-treated patients often received less reversal but still achieved outcomes comparable to or better than those with VKAs [4], supporting the interpretation that the intrinsic pharmacological properties of DOACs contribute to their safer bleeding profile. A multicentre observational study in patients on oral anticoagulants presenting with major haemorrhage added further perspective, demonstrating that plasma DOAC levels were rarely measured and frequently undetectable, yet reversal was administered in many cases. Despite such variation, mortality and hospital stay did not differ between patients treated with DOACs and those treated with VKAs [28]. Additionally, a retrospective cohort study from 35 trauma centres showed that in neurologically intact patients with minor traumatic ICH, reversal did not improve outcomes [29]. These findings suggest that once severe bleeding occurs, outcomes are determined less by the anticoagulant itself and more by neurological severity and timely system-level care, necessitating individualised reversal strategies based on neurological severity [29].

Neurosurgical interventions were infrequent in our cohort. The role of neurosurgical interventions in anticoagulant-associated ICH (AAICH) remains controversial and is recommended in a defined clinical scenario. A crucial, rapid reversal of anticoagulation is essential prior to operative intervention [30]. Most randomised trials have systematically excluded patients who are anticoagulated. Evidence from the International Surgical Trial in Intracerebral Haemorrhage, STICH I and II, failed to demonstrate an overall benefit of early craniotomy compared to best medical management [14,15]. Meta-analysis incorporating individual patient data suggested the potential benefit of early surgery in carefully selected cases, particularly when surgery is performed within 8 h, in lobar ICH volumes of 20–50 mL, and in patients with moderate GCS scores (9–12) [31]. A recent review emphasised that neurosurgical care alone is insufficient, and that outcomes improve most when integrated into a bundle of care that combines rapid reversal, blood pressure control, surgical selection, and high-quality stroke unit/ICU management [30,32,33].

This study provided detailed documentation of reversal strategies, systematic reporting of both short- and intermediate-term outcomes, and comparison with contemporary external data. The strengths of this study included real-world characterisation of reversal practice and inclusion of both spontaneous and traumatic ICH. However, we acknowledge some limitations concerning the present study, including the relatively small number of DOAC patients, which may limit power to detect subtle differences, a retrospective design that is prone to residual confounding, and a lack of follow-up imaging, which limited our ability to assess haematoma expansion, which would be a valuable addition for outcome assessment. Other important prognostic variables, namely, intraventricular extension and details of blood pressure management, were not incorporated. Moreover, the heterogeneity of reversal agents across the studied population might affect short- and long-term functional outcomes. Lastly, an imbalance in baseline demographics, such as older age in the DOAC group, could be addressed using a propensity score as appropriate when further studies with a larger sample size are conducted.

## 5. Conclusions

Our findings suggested that patients with DOAC-associated ICH had clinical outcomes comparable to those with VKA-associated ICH. Once haemorrhage occurs, long-term mortality and disability remain high regardless of anticoagulant type. These observations emphasise that the clinical focus should not only be on the choice of anticoagulant but also on prevention strategies, early recognition, standardised protocols for reversal and multidisciplinary care that integrates medical, surgical, and supportive management.

## Figures and Tables

**Table 1:** Baseline characteristics of participants with DOAC- and VKA-associated ICH.

Characteristics	Total (*n* = 171)	DOAC (*n* = 24)	VKA (*n* = 147)	*p* Value
Patient characteristics				
Age, years—mean (SD)	68.5 (12.7)	79.1 (9.6)	66.8 (12.4)	<0.001
Male—*n* (%)	83 (48.5)	13 (54.2)	70 (47.6)	0.55
BMI, kg/m^2^—mean (SD)	22.3 (3.8)	22.4 (4.3)	22.3 (3.7)	0.89
Comorbidities—*n* (%)				
Hypertension	90 (52.6)	19 (79.2)	71 (48.3)	0.007
Dyslipidaemia	60 (35.1)	13 (54.2)	47 (32.0)	0.04
Diabetes	45 (26.3)	8 (33.3)	37 (25.2)	0.40
Coronary heart disease	24 (14.0)	6 (25.0)	18 (12.2)	0.10
Congestive heart failure	20 (11.7)	1 (4.2)	19 (12.9)	0.32
Previous stroke	37 (21.6)	7 (29.2)	30 (20.4)	0.33
Chronic kidney disease	31 (18.1)	5 (20.8)	26 (17.7)	0.78
Admission information				
SBP, mmHg—mean (SD)	140.8 (26.7)	140.0 (36.7)	141.0 (24.8)	0.88
DBP, mmHg—mean (SD)	79.3 (19.3)	75.4 (16.5)	80.0 (19.7)	0.28
GCS—median (IQR)	14 (9, 15)	15 (12, 15)	14 (9, 15)	0.11
mRS—median (IQR)	3 (2, 4)	4 (3, 5)	3 (2, 4)	0.37
ATRIA Bleeding Risk Score—median (IQR)	3 (3, 6)	5 (3, 6)	3 (3, 5)	0.04
Medication—*n* (%)				
Indication of OAC				0.045
Atrial fibrillation	131 (76.6)	23 (95.8)	108 (73.5)	
Venous thromboembolism	4 (2.3)	0 (0)	4 (2.7)	
Valvular heart disease	29 (17.0)	0 (0)	29 (19.7)	
Others	7 (4.1)	1 (4.2)	6 (4.1)	
VKA dose, mg/week—mean (SD)	20.9 (9.2)	NA	20.9 (9.2)	NA
Concurrent antiplatelet therapy	10 (5.9)	2 (8.3)	8 (5.4)	0.63
Laboratory—mean (SD)				
Haemoglobin, g/dL	11.1 (2.3)	11.4 (2.3)	11.1 (2.3)	0.61
Platelet count, cells/mm^3^	219,680 (89,424)	233,667 (102,550)	217,365 (87,245)	0.41
Creatinine, mg/dL	1.4 (1.7)	1.1 (0.4)	1.5 (1.8)	0.34
eGFR, mL/min/1.72 m^2^	64.1 (29.4)	59.1 (21.5)	64.8 (30.4)	0.41
INR	3.2 (3.2)	1.6 (0.8)	3.5 (3.4)	0.008

Abbreviations: ATRIA, Anticoagulation and Risk Factors in Atrial Fibrillation; BMI, body mass index; DBP, diastolic blood pressure; DOAC, direct oral anticoagulant; eGFR, estimated glomerular filtration rate; GCS, Glasgow Coma Scale; ICH, intracranial haemorrhage; INR, international normalised ratio; IQR, interquartile range; mRS, modified Rankin Scale; NA, not applicable; OAC, oral anticoagulant; SBP, systolic blood pressure; SD, standard deviation; VKA, vitamin K antagonist.

**Table 2:** ICH characteristics and management.

	Total (*n* = 171)	DOAC (*n* = 24)	VKA (*n* = 147)	*p* Value
Traumatic ICH—*n* (%)	86 (50.3)	18 (75.0)	68 (46.3)	0.009
ICH location—*n* (%)				0.09
Intracerebral haemorrhage	19 (11.1)	3 (12.5)	16 (10.9)	
Subdural haematoma	128 (74.9)	14 (58.3)	114 (77.6)	
Epidural haematoma	2 (1.2)	1 (4.2)	1 (0.7)	
Subarachnoid haemorrhage	5 (2.9)	1 (4.2)	4 (2.7)	
Mixed location	17 (9.9)	5 (20.8)	12 (8.1)	
CHARTS—*n* (%)				0.17
Lobar	16 (84.2)	2 (66.7)	14 (87.5)	
Deep and infratentorial	1 (5.3)	1 (33.3)	0 (0)	
Uncertain	2 (10.5)	0 (0)	2 (12.5)	
Intracerebral haemorrhage volume, mL—mean (SD)	5.4 (23.7)	13.9 (47.3)	4.0 (17.0)	0.06
Intracerebral haemorrhage volume, mL—mean (95% CI) *	30.6 (18.7–56.6)	58.3 (37.5–82.6)	27.9 (15.9–49.2)	<0.001
Neurosurgical interventions—*n* (%)				0.03
None	84 (49.1)	10 (41.7)	74 (50.3)	
Intracerebral haemorrhage removal	40 (23.4)	2 (8.3)	38 (25.9)	
Extra ventricular drain	29 (17.0)	7 (29.2)	22 (15.0)	
Burr hole to drain	18 (10.5)	5 (20.8)	13 (8.8)	
Reversal agents †—*n* (%)				
None	32 (18.7)	8 (33.3)	24 (16.3)	0.05
Vitamin K	62 (36.3)	1 (4.2)	61 (41.5)	<0.001
Tranexamic acid	13 (7.6)	0 (0)	13 (8.8)	0.22
Fresh frozen plasma	124 (72.5)	8 (33.3)	116 (78.9)	<0.001
Prothrombin complex concentrate	12 (7.0)	7 (29.2)	5 (3.4)	<0.001
Idarucizumab	5 (2.9)	5 (20.8)	0 (0)	<0.001
rFVIIa	3 (1.8)	0 (0)	3 (2.0)	1.00
Thrombotic complications—*n* (%)	7 (4.1)	0 (0)	7 (4.8)	0.60
Length of stay, days—median (IQR)	11 (6, 18)	10 (5, 14)	11 (6, 18)	0.30

Abbreviations: CI, confidence interval; DOAC, direct oral anticoagulant; ICH, intracranial haemorrhage; IQR, interquartile range; CHARTS, Cerebral Haemorrhage Anatomical RaTing inStrument; rFVIIa, recombinant factor VIIa; SD, standard deviation; VKA, vitamin K antagonist. * Because of the significant skewness of intracerebral haemorrhage volume, further geometric means using log transformation were utilised. † The total percentage exceeded 100 due to some patients receiving more than one reversal agent.

**Table 3:** Study outcomes using oral anticoagulants.

Outcomes	Event Rate, No./Total No. (%)	Unadjusted OR (95% CI)	*p* Value	Model 1 Adjusted OR (95% CI) ^†^	*p* Value	Model 2 Adjusted OR (95% CI) ^‡^	*p* Value
In-hospital outcomes							
Mortality							
DOAC	5/24 (20.8)	1.58 (0.41–5.02)	0.41	0.75 (0.20–2.87)	0.68	1.30 (0.39–4.36)	0.67
VKA	21/147 (14.3)	1 (Reference)	NA	1 (Reference)	NA	1 (Reference)	NA
Independent ambulation at discharge							
DOAC	8/24 (33.3)	0.52 (0.18–1.39)	0.15	0.92 (0.33–2.58)	0.87	0.88 (0.32–2.40)	0.80
VKA	72/147 (49.0)	1 (Reference)	NA	1 (Reference)	NA	1 (Reference)	NA
90-day outcomes							
Mortality							
DOAC	7/24 (29.2)	1.75 (0.56–4.97)	0.25	0.96 (0.29–3.13)	0.94	1.20 (0.41–3.53)	0.74
VKA	28/147 (19.1)	1 (Reference)	NA	1 (Reference)	NA	1 (Reference)	NA
mRS 0–1							
DOAC	7/24 (29.2)	0.48 (0.16–1.31)	0.12	1.00 (0.35–2.85)	1.00	0.83 (0.29–2.34)	0.72
VKA	68/147 (46.3)	1 (Reference)	NA	1 (Reference)	NA	1 (Reference)	NA
mRS 0–2							
DOAC	12/24 (50.0)	0.69 (0.26–1.81)	0.40	1.39 (0.51–3.77)	0.51	0.89 (0.33–2.41)	0.83
VKA	87/147 (59.2)	1 (Reference)	NA	1 (Reference)	NA	1 (Reference)	NA

Abbreviations: CI, confidence interval; DOAC, direct oral anticoagulant; mRS, modified Rankin Scale; NA, not applicable; OR, odds ratio; VKA, vitamin K antagonist. ^†^ Adjusted for age, hypertension, intracranial haemorrhage aetiology, intracerebral haemorrhage volume, and indication of oral anticoagulant. ^‡^ Adjusted for age, hypertension, treatment (neurosurgical interventions and reversal agents), and indication of oral anticoagulant.

## Data Availability

The datasets generated and/or analysed during the current study are available from the corresponding author on reasonable request.

## Supplementary Materials

The following supporting information can be downloaded at: https://www.mdpi.com/article/10.3390/clinpract16040079/s1, Table S1: Details of DOAC use and thrombotic complications; Table S2. Post hoc power calculation for the study outcomes; Table S3. Stratified analyses according to ICH aetiology.

## References

[1] Lopes R.D., Guimarães P.O., Kolls B.J., Wojdyla D.M., Bushnell C.D., Hanna M., Easton J.D., Thomas L., Wallentin L., Al-Khatib S.M. (2017). Intracranial hemorrhage in patients with atrial fibrillation receiving anticoagulation therapy. Blood J. Am. Soc. Hematol..

[2] Estevez-Fraga C., Molina-Sanchez M., Alvarez-Velasco R., Agüero-Rabes P., Crespo-Araico L., Viedma-Guiard E., Cruz-Culebras A., Matute C., Vera R., De Felipe-Mimbrera A. (2018). Quality of Chronic Anticoagulation Control in Patients with Intracranial Haemorrhage due to Vitamin K Antagonists. Stroke Res. Treat..

[3] Wu T., Lv C., Wu L., Chen W., Lv M., Jiang S., Zhang J. (2022). Risk of intracranial hemorrhage with direct oral anticoagulants: A systematic review and meta-analysis of randomized controlled trials. J. Neurol..

[4] Pfeilschifter W., Lindhoff-Last E., Alhashim A., Zydek B., Lindau S., Konstantinides S., Grottke O., Nowak-Göttl U., von Heymann C., Birschmann I. (2022). Intracranial bleeding under vitamin K antagonists or direct oral anticoagulants: Results of the RADOA registry. Neurol. Res. Pract..

[5] Kawabori M., Niiya Y., Iwasaki M., Mabuchi S., Ozaki H., Matsubara K., Houkin K. (2018). Characteristics of symptomatic intracerebral hemorrhage in patient receiving direct oral anticoagulants: Comparison with warfarin. J. Stroke Cerebrovasc. Dis..

[6] Inohara T., Xian Y., Liang L., Matsouaka R.A., Saver J.L., Smith E.E., Schwamm L.H., Reeves M.J., Hernandez A.F., Bhatt D.L. (2018). Association of intracerebral hemorrhage among patients taking non–vitamin K antagonist vs vitamin K antagonist oral anticoagulants with in-hospital mortality. JAMA.

[7] Kurogi R., Nishimura K., Nakai M., Kada A., Kamitani S., Nakagawara J., Toyoda K., Ogasawara K., Ono J., Shiokawa Y. (2018). Comparing intracerebral hemorrhages associated with direct oral anticoagulants or warfarin. Neurology.

[8] Chai-Adisaksopha C., Hillis C., Isayama T., Lim W., Iorio A., Crowther M. (2015). Mortality outcomes in patients receiving direct oral anticoagulants: A systematic review and meta-analysis of randomized controlled trials. J. Thromb. Haemost..

[9] Hagii J., Tomita H., Metoki N., Saito S., Shiroto H., Hitomi H., Kamada T., Seino S., Takahashi K., Baba Y. (2014). Characteristics of intracerebral hemorrhage during rivaroxaban treatment: Comparison with those during warfarin. Stroke.

[10] Tsivgoulis G., Lioutas V.-A., Varelas P., Katsanos A.H., Goyal N., Mikulik R., Barlinn K., Krogias C., Sharma V.K., Vadikolias K. (2017). Direct oral anticoagulant–vs vitamin K antagonist–related nontraumatic intracerebral hemorrhage. Neurology.

[11] Adachi T., Hoshino H., Takagi M., Fujioka S., Saiseikai Stroke Research Group (2017). Volume and characteristics of intracerebral hemorrhage with direct oral anticoagulants in comparison with warfarin. Cerebrovasc. Dis. Extra.

[12] Wilson D., Seiffge D.J., Traenka C., Basir G., Purrucker J.C., Rizos T., Sobowale O.A., Sallinen H., Yeh S.-J., Wu T.Y. (2017). Outcome of intracerebral hemorrhage associated with different oral anticoagulants. Neurology.

[13] Foerch C., Lo E.H., van Leyen K., Lauer A., Schaefer J.H. (2019). Intracerebral Hemorrhage Formation Under Direct Oral Anticoagulants: Clinical and Translational Evidence. Stroke.

[14] Mendelow A.D., Gregson B.A., Fernandes H.M., Murray G.D., Teasdale G.M., Hope D.T., Karimi A., Shaw M.D.M., Barer D.H. (2005). Early surgery versus initial conservative treatment in patients with spontaneous supratentorial intracerebral haematomas in the International Surgical Trial in Intracerebral Haemorrhage (STICH): A randomised trial. Lancet.

[15] Mendelow A.D., Gregson B.A., Rowan E.N., Murray G.D., Gholkar A., Mitchell P.M. (2013). Early surgery versus initial conservative treatment in patients with spontaneous supratentorial lobar intracerebral haematomas (STICH II): A randomised trial. Lancet.

[16] Charidimou A., Schmitt A., Wilson D., Yakushiji Y., Gregoire S.M., Fox Z., Jäger H.R., Werring D.J. (2017). The Cerebral Haemorrhage Anatomical RaTing inStrument (CHARTS): Development and assessment of reliability. J. Neurol. Sci..

[17] Kothari R.U., Brott T., Broderick J.P., Barsan W.G., Sauerbeck L.R., Zuccarello M., Khoury J. (1996). The ABCs of measuring intracerebral hemorrhage volumes. Stroke.

[18] Ahmed A., Ahmed R., Ali S.S., Patel U., Shahid I., Zafar M., Sharma A., Ashraf A., Jani V. (2020). Intracerebral hemorrhage outcomes in patients using direct oral anticoagulants versus vitamin K antagonists: A meta-analysis. Clin. Neurol. Neurosurg..

[19] Gerner S.T., Kuramatsu J.B., Sembill J.A., Sprügel M.I., Hagen M., Knappe R.U., Endres M., Haeusler K.G., Sobesky J., Schurig J. (2019). Characteristics in non–vitamin K antagonist oral anticoagulant–related intracerebral Hemorrhage. Stroke.

[20] Chen S.-J., Yeh S.-J., Tang S.-C., Lin S.-Y., Tsai L.-K., Jeng J.-S. (2020). Similar outcomes between vitamin K and non-vitamin K antagonist oral anticoagulants associated intracerebral hemorrhage. J. Formos. Med. Assoc..

[21] Yu J.-H., Li P.-R., Chen D.-Y., Huang W.-K., See L.-C. (2024). Mortality after major bleeding in Asian atrial fibrillation patients receiving different direct oral anticoagulants: A nationwide, propensity score study. Sci. Rep..

[22] Liu Y.-L., Yin L., Gu H.-M., Zhu X.-J., Huang X.-X. (2022). Outcomes of elderly patients with traumatic brain injury associated with the pre-injury antithrombotic prophylaxis type—A systematic review and meta-analysis. Eur. Rev. Med. Pharmacol. Sci..

[23] Batey M., Hecht J., Callahan C., Wahl W. (2018). Direct oral anticoagulants do not worsen traumatic brain injury after low-level falls in the elderly. Surgery.

[24] Siepen B.M., Forfang E., Branca M., Drop B., Mueller M., Goeldlin M.B., Katan M., Michel P., Cereda C., Medlin F. (2024). Intracerebral haemorrhage in patients taking different types of oral anticoagulants: A pooled individual patient data analysis from two national stroke registries. Stroke Vasc. Neurol..

[25] Duquet-Armand M., Bouziane A., Arruda A., Denault J.-S., Routhier A., Cusson T., Halwagi A.E., Williamson D., Perreault M., Lordkipanidze M. (2023). Spontaneous Intracranial Hemorrhage and Oral Anticoagulation: A Retrospective Study. Can. J. Gen. Intern. Med..

[26] Grottke O., Afshari A., Ahmed A., Arnaoutoglou E., Bolliger D., Fenger-Eriksen C., von Heymann C. (2024). Clinical guideline on reversal of direct oral anticoagulants in patients with life threatening bleeding. Eur. J. Anaesthesiol..

[27] Frontera J.A., Lewin J.J., Rabinstein A.A., Aisiku I.P., Alexandrov A.W., Cook A.M., del Zoppo G.J., Kumar M.A., Peerschke E.I., Stiefel M.F. (2016). Guideline for reversal of antithrombotics in intracranial hemorrhage: A statement for healthcare professionals from the Neurocritical Care Society and Society of Critical Care Medicine. Neurocritical Care.

[28] Baker R.I., Gilmore G., Chen V., Young L., Merriman E., Curnow J., Joseph J., Tiao J.Y., Chih J., McRae S. (2023). Direct oral anticoagulants or vitamin K antagonists in emergencies: Comparison of management in an observational study. Res. Pract. Thromb. Haemost..

[29] Powell K., Curtiss W., Sadek E., Hecht J. (2024). Is reversal of anticoagulants necessary in neurologically intact traumatic intracranial hemorrhage?. Pharmacother. J. Hum. Pharmacol. Drug Ther..

[30] Le Roux P., Pollack C.V., Milan M., Schaefer A. (2014). Race against the clock: Overcoming challenges in the management of anticoagulant-associated intracerebral hemorrhage. J. Neurosurg..

[31] Gregson B.A., Broderick J.P., Auer L.M., Batjer H., Chen X.-C., Juvela S., Morgenstern L.B., Pantazis G.C., Teernstra O.P., Wang W.-Z. (2012). Individual patient data subgroup meta-analysis of surgery for spontaneous supratentorial intracerebral hemorrhage. Stroke.

[32] Morgenstern L.B., Hemphill J.C., Anderson C., Becker K., Broderick J.P., Connolly E.S., Greenberg S.M., Huang J.N., Macdonald R.L., Messé S.R. (2010). Guidelines for the management of spontaneous intracerebral hemorrhage: A guideline for healthcare professionals from the American Heart Association/American Stroke Association. Stroke.

[33] Parry-Jones A.R., Moullaali T.J., Ziai W.C. (2020). Treatment of intracerebral hemorrhage: From specific interventions to bundles of care. Int. J. Stroke.

