# Supplementary material for: Outcomes of Intracranial Haemorrhage in Patients Taking Direct Oral Anticoagulants or Vitamin K Antagonists: A Seven-Year Single-Centre Retrospective Analysis
Source: Clin Pract. 2026 Apr 18;16(4):79. doi: 10.3390/clinpract16040079 (PMC13115525; doi:10.3390/clinpract16040079)
Supplement: Supplementary file 1 [file clinpract-16-00079-s001.zip › clinpract-4191333-supplementary.pdf]

Outcomes of Intracranial Haemorrhage in Patients Taking Direct Oral Anticoagulants or Vitamin K Antagonists: A Seven-Year Single-Centre Retrospective Analysis

Supplementary material:

- **Table S1.** Details of DOAC use and thrombotic complications.
- **Table S2.** Post hoc power calculation for the study outcomes.
- **Table S3.** Stratified analyses according to ICH aetiology.

Table S1. Details of DOAC use and thrombotic complications.

	**Total (n=171)**	**DOAC (n=24)**	**VKA (n=147)**	***p* value**
DOAC – n (%)				
Dabigatran		9 (37.5)		NA
Rivaroxaban		6 (25.0)		
Apixaban		4 (16.7)		
Edoxaban		5 (20.8)		
Thrombotic complications – n (%)				1.00
None	164 (96.0)	24 (100.0)	140 (95.3)	
Myocardial infarction	1 (0.6)	0 (0)	1 (0.7)	
Stroke	3 (1.7)	0 (0)	3 (2.0)	
Others	3 (1.7)	0 (0)	3 (2.0)	

Abbreviations: DOAC, direct oral anticoagulant; NA, not applicable; VKA, vitamin K antagonist.

Table S2. Post hoc power calculation for the study outcomes.

Study outcomes	Actual sample sizes	Proportions		Estimated power
		DOAC	VKA	
In-hospital mortality	170	0.21	0.14	0.22
Independent ambulation at discharge	170	0.33	0.49	0.56
90-day mortality	170	0.29	0.19	0.33
90-day mRS 0-1	170	0.29	0.46	0.63
90-day mRS 0-2	170	0.50	0.59	0.22

Abbreviations: DOAC, direct oral anticoagulant; mRS, modified Rankin Scale; VKA, vitamin K antagonist.

Table S3. Stratified analyses according to ICH aetiology.

Study outcomes	Non-traumatic			Traumatic		
	**OR**	**95% CI**	***p* value**	**OR**	**95% CI**	***p* value**
In-hospital mortality	0.65	0.03-12.5	0.77	1.67	0.53-5.32	0.38
Independent ambulation at discharge	0.38	0.08-1.90	0.24	0.89	0.31-2.58	0.83
90-day mortality	0.46	0.02-8.70	0.60	1.92	0.66-5.58	0.23
90-day mRS 0-1	0.24	0.04-1.54	0.13	0.83	0.29-2.42	0.74
90-day mRS 0-2	1.72	0.27-11.08	0.57	0.69	0.25-1.94	0.48

Abbreviations: CI, confidence interval; ICH, intracranial haemorrhage; mRS, modified Rankin Scale; OR, odds ratio.
